# Supplementary material for: ZNF143 binds DNA and stimulates transcripstion initiation to activate and repress direct target genes
Source: bioRxiv. 2024 May 15:2024.05.13.594008. Preprint. [Version 1] doi: 10.1101/2024.05.13.594008 (PMC11118474; doi:10.1101/2024.05.13.594008)
Supplement: Supplement 1 [file NIHPP2024.05.13.594008v1-supplement-1.pdf]

bioRxiv preprint doi: https://doi.org/10.1101/2024.05.13.594008; this version posted May 15, 2024. The copyright holder for this preprint (which was not certified by peer review) is the author/funder, who has granted bioRxiv a license to display the preprint in perpetuity. It is made available under aCC-BY 4.0 International license.

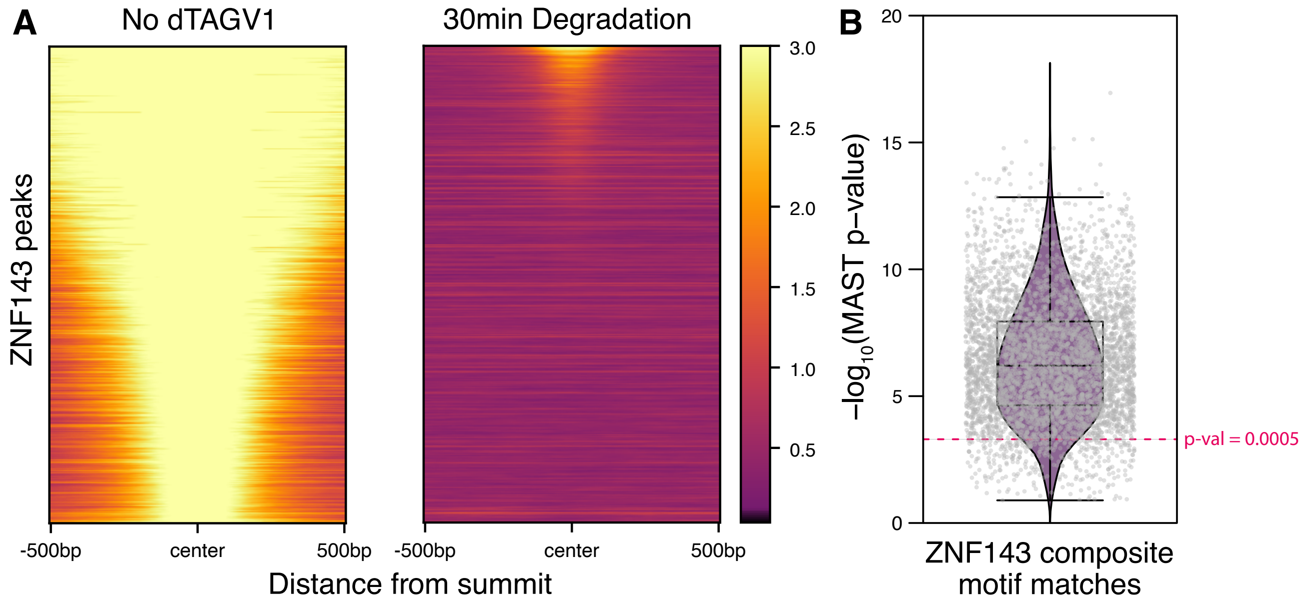

Fig. S1. ZNF143 is rapidly depleted from chromatin after 30 minutes of dTAG treatment and we precisely define the ZNF143 binding site with motif analysis. Motif analysis found that all ZNF143 ChIP-seq peaks have a ZNF143 sequence that conforms to the Figure 1D composite motif with a p-value of 0.13 or less, with 93% of peaks at a p-value less than 0.0005.

bioRxiv preprint doi: https://doi.org/10.1101/2024.05.13.594008; this version posted May 15, 2024. The copyright holder for this preprint (which was not certified by peer review) is the author/funder, who has granted bioRxiv a license to display the preprint in perpetuity. It is made available under aCC-BY 4.0 International license.

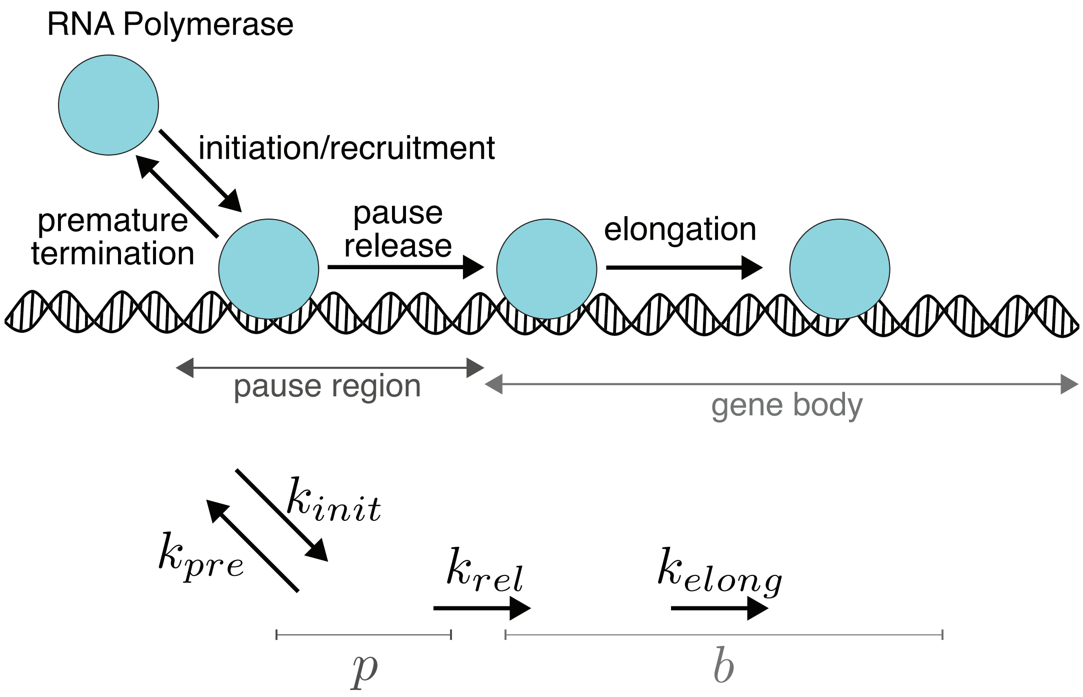

$$\frac{dp}{dt} = k_{init} - (k_{pre} + k_{rel})\, p$$

$$\frac{db}{dt} = k_{rel}\, p - k_{elong}\, b$$

Fig. S2. Compartment model for PRO-seq densities. This model and illustration was adapted from our previous work (Dutta et al. 2023). The pause densities p and body densities b are calculated directly from normalized PRO-seq data and elongation rates are constrained to a rate of 2-4kb/minute. The other parameters are calculated from the model.

bioRxiv preprint doi: https://doi.org/10.1101/2024.05.13.594008; this version posted May 15, 2024. The copyright holder for this preprint (which was not certified by peer review) is the author/funder, who has granted bioRxiv a license to display the preprint in perpetuity. It is made available under aCC-BY 4.0 International license.

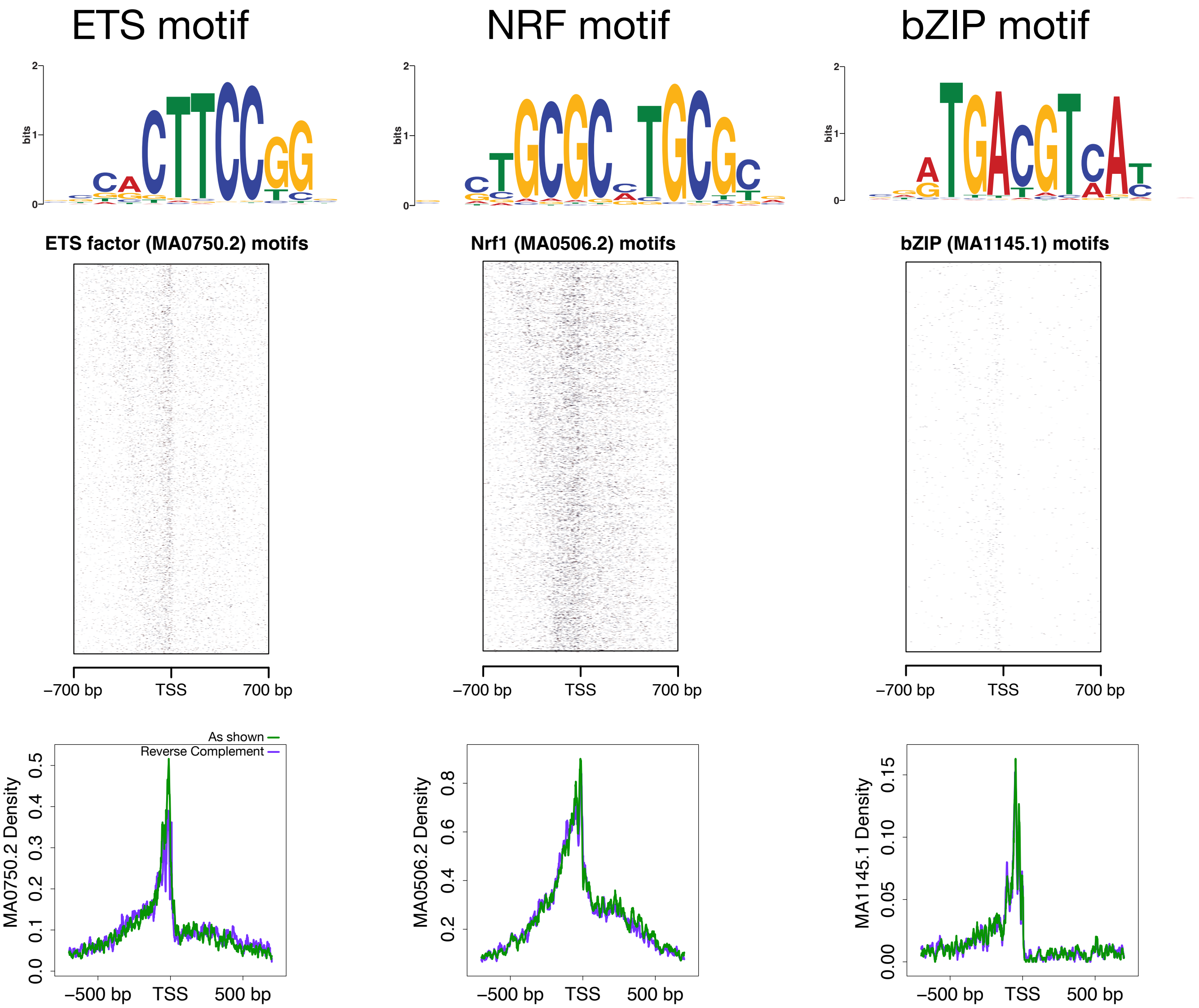

Fig. S3. ETS, NRF, and bZIP motifs are enriched in promoters. *De novo* motif analysis within promoters revealed several motifs enriched between the same divergent and sense TSSs shown in Figure 7A. We used the best match to the JASPAR database (seqLogos) for each motif to identify their locations relative to TSSs with FIMO. The density plots quantify the distribution of the seqLogo-illustrated motif (top) and the reverse complement motif relative to TSSs.

bioRxiv preprint doi: https://doi.org/10.1101/2024.05.13.594008; this version posted May 15, 2024. The copyright holder for this preprint (which was not certified by peer review) is the author/funder, who has granted bioRxiv a license to display the preprint in perpetuity. It is made available under aCC-BY 4.0 International license.

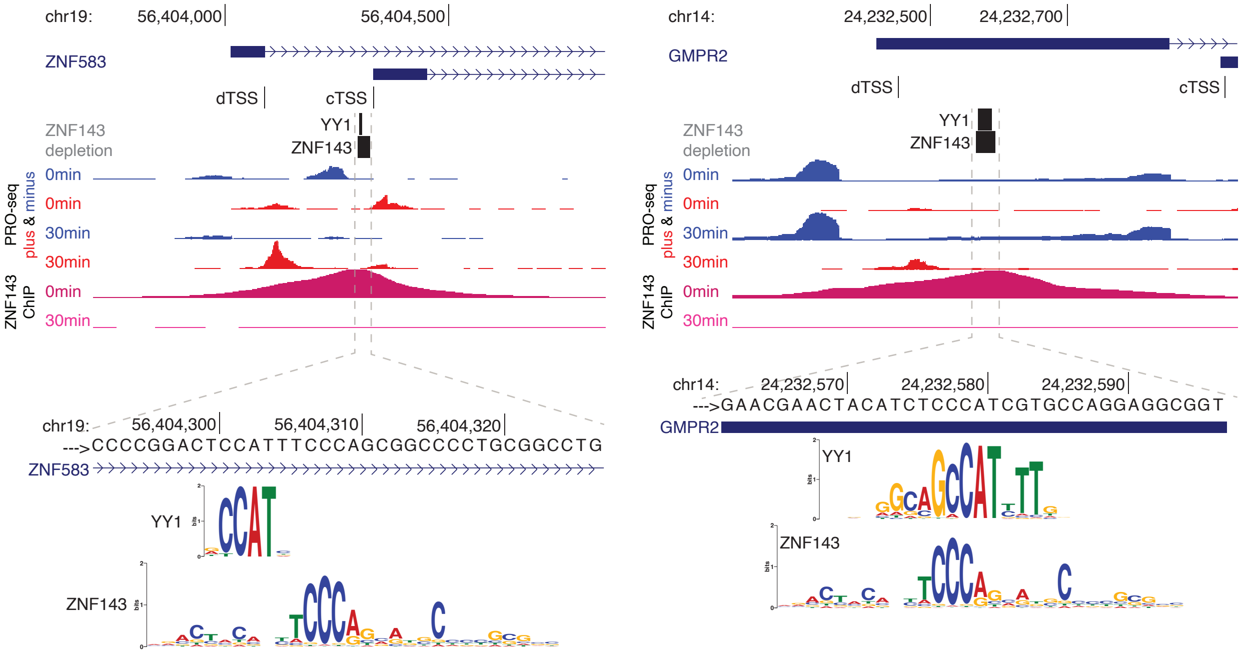

Fig. S4. ZNF143 may block YY1 to reduce gene expression. The max TSS position of *ZNF583* and *GMPR2* shift upstream, suggesting a mechanism whereby YY1 access after ZNF143 degradation facilitates initiation at the dTAG TSS (cTSS: control max TSS position. dTSS: dTAG max TSS position).

bioRxiv preprint doi: https://doi.org/10.1101/2024.05.13.594008; this version posted May 15, 2024. The copyright holder for this preprint (which was not certified by peer review) is the author/funder, who has granted bioRxiv a license to display the preprint in perpetuity. It is made available under aCC-BY 4.0 International license.

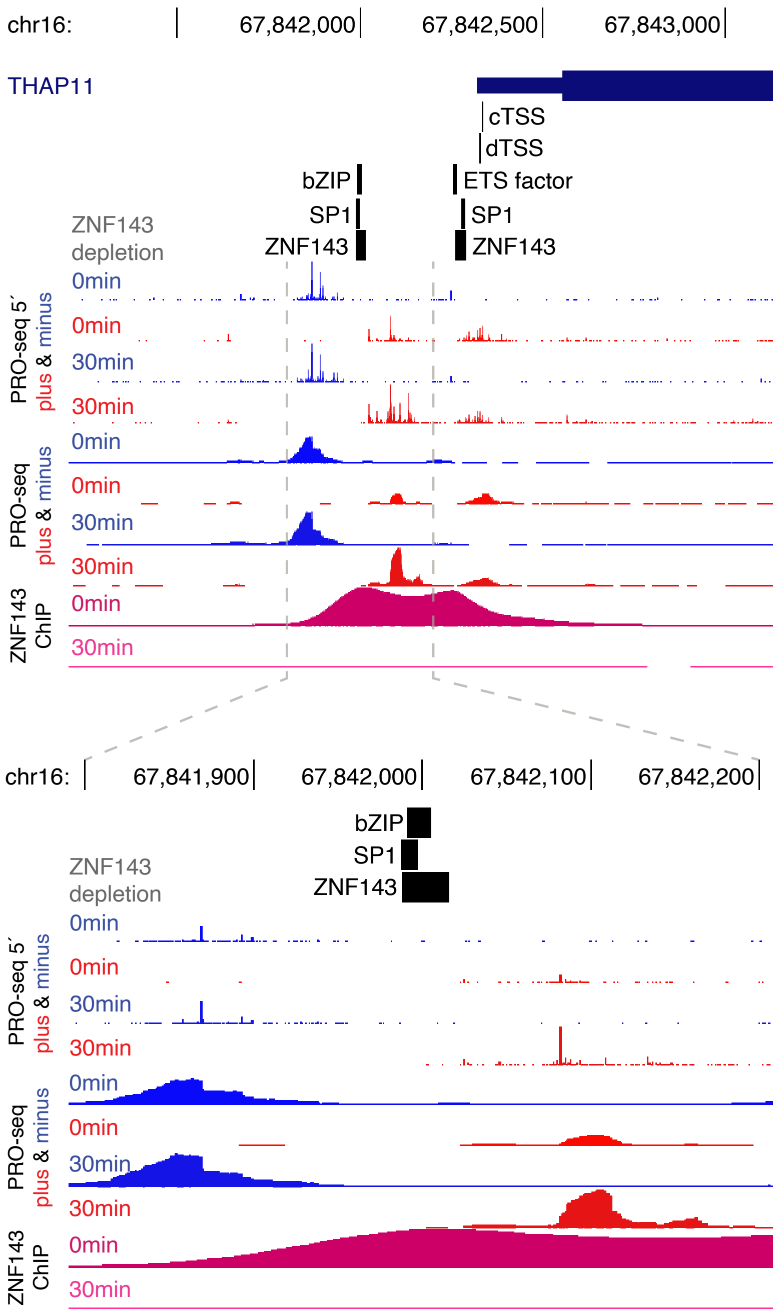

Fig. S5. ZNF143 regulates *Thap11* via a complex mechanism. Depletion of ZNF143 allows for greater usage of a TSS upstream of both the control and dTAG TSSs. ZNF143 may repress transcription of *Thap11* by displacing other factors, such as ETS or a bZIP factor, that may be more potent activators of *Thap11*. Note that the ZNF143 motif shown in Figure 7D is most proximal to the TSS, but an upstream ZNF143 binding site/motif overlaps a bZIP motif. We do not conclude that SP1 redistributes because we did not observe a notable change in SP1 ChIP peak intensity.
